# Incorporation of Optical Density into the Blending Design for a Biocement Solution

**DOI:** 10.3390/ma15051951

**Published:** 2022-03-06

**Authors:** Masaharu Fukue, Zbigniew Lechowicz, Yuichi Fujimori, Kentaro Emori, Catherine N. Mulligan

**Affiliations:** 1 Japanese Geotechnical Association for Housing Disaster Prevention, 1622, Oshikiri, Shimizu-ku, Shizuoka 424-0008, Japan; fukue@scc.u-tokai.ac.jp; 2 Department of Geotechnical Engineering, Institute of Civil Engineering, Warsaw University of Life Sciences, Nowoursynowska 159, 02-776 Warsaw, Poland; 3 Chubu Sokuchi Research Institute Co., 801-1 Konami, Suwa 392-0131, Japan; fujimori-yuichi@chubusokuchi-lab.co.jp; 4 Sanko Kaihatsu Co., Ltd., 1320 Gokanjima, Fuji 416-0946, Japan; k.emori@sankoukaihathu.co.jp; 5 Department of Building, Civil and Environment Engineering, Concordia University, 1455 de Maisonneuve Blvd. W., Montreal, QC H3G 1M8, Canada; mulligan@civil.concordia.ca

**Keywords:** urease, standard precipitation of carbonates, optical density, viability of cells, blending design

## Abstract

The engineering practices for applying the microbial precipitation of carbonates require a design of the blending biocement solution (BCS). The BCS is usually blended with concentrated strains NO-A10, reaction media, such as urea and CaCl_2_, and a solvent, i.e., water or seawater. To characterize the BCS, the unknown microbial characteristics, such as the cell viability, are complex factors. Therefore, the optical density (OD) was redefined as Rcv OD*, in which OD* was the tentative OD of the BCS used and Rcv was the conversion rate concerning the cell viability. To determine Rcv values, a standard precipitation curve based on the precipitation rate at 24 h was determined. It was found that the curve was expressed by λ_1_ OD+ λ_2_ OD^2^, in which λ_1_ and λ_2_ were 8.46 M and −17.633 M, respectively. With this, the Rcv and OD values of unknown BCS were estimated from the results of precipitation tests using arbitrary OD* values. By extending the testing time, the second order term of OD or OD* was negligible. Accordingly, the precipitation amount is expressed as 8.46 OD, in which the OD can be estimated by precipitation tests using arbitrary OD* values of BCSs. Unless the Ca^2+^ value is dominant, the optimum blending of BCS can be determined by OD. Thus, it is concluded that the blending design of BCS is achieved using 8.46 OD, or 8.46 Rcv OD*, and the standard precipitation curve was defined in this study.

## 1. Introduction

The cementation process of natural sediments by carbonates is called carbonate diagenesis. The carbonates existing in the sediments are attributed to the buried effect of dead foraminifers and coccoliths [1,2,3,4] or biomineralization [5,6]. It was shown that natural deposited marine soils exhibited significant relationships between unconfined compressive strength and calcium carbonate contents [3]. The results showed that the silty surface sediments at depths of up to 6 m below the sea bottom in Tokyo Bay and Osaka Bay showed increasing rates of 7.5 kPa vane shear strength per a 1.0% increase in carbonate content. On the other hand, silty sediments at a depth of approximately 24 m below the sea bottom off Haneda Airport showed an increase in unconfined compressive strength of 64 kPa per 1.0% carbonate content. Since then, improvement in the carbonation technique in soil has been desired.

For example, Mobley and Hausinger [7] illustrated microbial ureases in terms of their significance, regulation, and molecular characterization, and contributed to expanding their medical knowledge on urease to a wide range of readers in other fields. On the other hand, subsequent chemical reactions in carbonate production after urease activity can be seen in agricultural papers [8]. Carbonate precipitation has been used to inhibit the loss of ammonia as fertilizer to the air and to retain the ammonium ions in the soil.

Carbonate diagenesis is distinguished from microbially precipitated carbonates because of natural processes. However, the results and evaluation in engineering may have similarities. The microbial precipitation of carbonate using ureolytic activity has been increasingly studied worldwide and the objectives of studies have been expanding [9,10,11,12,13,14,15,16,17,18]. These are based on microbially induced carbonate precipitation and are often called MICP techniques.

In the field of soil improvement, there may be no doubt that the most dominant factors are the carbonate content induced microbially and the soil characteristics. Other factors are environmental conditions, such as temperature, pH, etc. For example, it is known that the mechanical strength of soils has been shown in terms of carbonate content [19]. Van Paassen [20] investigated the microbially induced carbonate solidification of medium- to high-density sands. The study showed that the UCS, deviatoric stress, and tensile strength increased exponentially with the CaCO_3_ content and initial density of the specimens. Non-destructive measurements were also applied to investigate the carbonate-cementing effects [21,22]. Fukue and Lechowicz [23] showed that, using the pocket penetrometer, the microbially induced carbonate content was the most dominant factor in controlling the strength development of sandy soils. They found that, for loose to medium density sands, the unconfined compressive strength (UCS) estimated by measurements was expressed as a linear function of the carbonate content. It was also shown that the proportional constants varied with the soil type, such as the grain size, distribution, and initial density. Similar results have been reported by many studies [24].

Thus, regardless of whether it is natural or artificial, the evaluation of microbial carbonate precipitation can be achieved with ultimate amounts of induced carbonates. However, it is required to predict or design the ultimate amounts of induced carbonates for engineering practice.

In nature, urease (enzyme) is produced by many organisms, such as animals and plants, including microorganisms. The enzyme activity, i.e., ureolysis due to the urease, is well known as:

(NH_2_)_2_CO + [e] + H_2_O → 2NH_3_ + CO_2_ (1)

**Figure 1:**
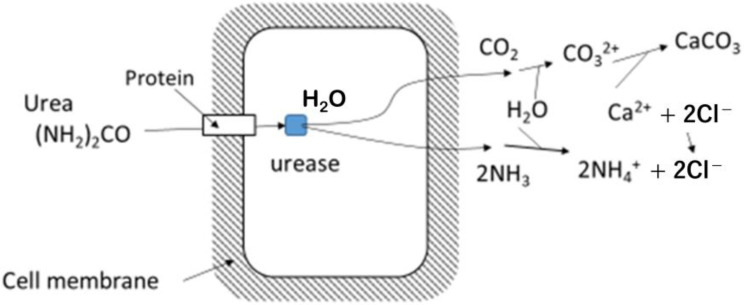
Microbial hydrolysis of urea, showing that first products are CO_2_ and NH_3_ inside the microbe’s cell and carbonates are produced outside of the cell.

Reaction (1) is known as hydrolysis with the catalysis of urease [e]. A microbe’s system is shown in Figure 1, in which, the first products are NH_3_ and CO_2_ [7]. The 2NH_3_ and CO_2_ can easily be transported outside of cells, and react with H_2_O as follows:

2NH_3_ + CO_2_ + H_2_O → 2(NH_4_)^+^ + CO_3_^2−^ (2)

It should be remembered that the enzyme [e] in Reaction (1) is not given appropriately in terms of quantity and quality. The species and strains of ureolytic microbes, their concentrations, and their micro-environments play an important role for the urease activity [7,13,15,25,26]. For example, the urease activity of biomass increases proportionally to the numbers of cells [25]. This implies that the concentration of microbes plays an important role in the generation rate of carbonate ions.

The product (NH_4_)_2_ CO_3_·H_2_O increases the pH outside of the cell [8] and helps Reaction (3) in the presence of Ca^2+^. Reaction (3) indicates that 1 M CaCO_3_ can be produced when 1 M urea and 1M CaCl_2_ are used, as long as no inhibition of urease activity occurs in Reaction (1). Accordingly, the concentration of Ca^2+^ becomes a dominant factor for the CaCO_3_ precipitation rate.

Ca^2+^ + CO_3_^2−^ → CaCO_3_ (3)

The production of CaCO_3_ does not change the pH of the system. The Ca^2+^ is usually supplied in the form of calcium chloride, CaCl_2_. Then, NH_4_^+^ and Cl^−^ may increase in the system, and the pH of the solution decreases during Reaction (3).

For the improvement of soils, rock, and concrete using microbial carbonate precipitation, there are basically three problems: the reliability, repeatability, and cost performance. This may result from the drawback in using microbes whose properties and characteristics are influenced by the change in cell viability. This problem becomes serious directly or indirectly when scale-up and commercial applications are considered [27]. In many studies, bacteria and media have been injected into soils for in situ cultivation. In those cases, bacteria cultivation has not always been well governed, and the design of the blending biocement solution (BCS) cannot be made. Under this condition, the reliability and repeatability are difficult to obtain. Therefore, the aim of this study is to solve the problem concerning the unknown quality of the BCS using stored frozen microbes.

## 2. Materials and Methods

### 2.1. Microbial Preparation

In this study, microbes were cultivated using a cell incubator (Life-Engineering, Shizuoka, Japan) and prepared with a continuous supply system. This may enable an optimum design of the blending of BCS. The culture solution is centrifuged, and the harvest is stored in a deep freezer (Life-Engineering, Shizuoka, Japan). With these processes, the volume reduction, the long-term-storage, easy and cheap transportation, etc., are achieved. At the same time, the cost performance is improved with the new development of cultivation techniques. The concentration of microorganisms drastically increased up to an optical density (OD_600_) of 4.0 by developing a good medium for the bacteria. Therefore, a smaller cell incubator was enabled.

In 2008, the ureolytic microbes used in this study were isolated from the alluvial soils in Japan. The microbe strains were named NO-A10 [28]. The microbes were cultivated in a cell incubator with a volume of 1 m^3^. At present, the culture volume for a batch process is usually 300 L. 

**Figure 2:**
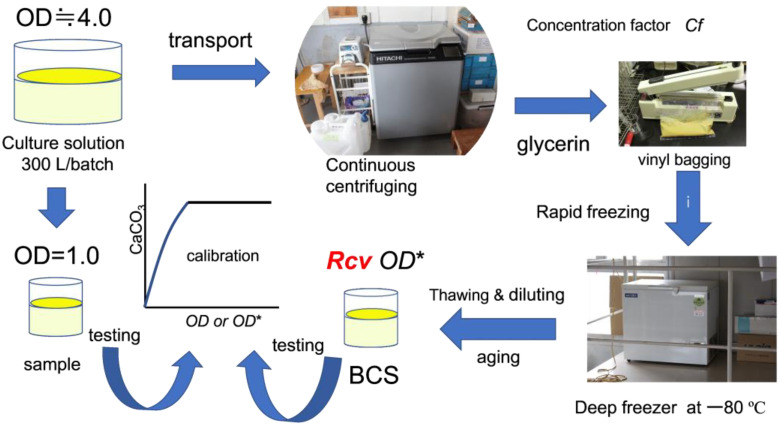
From culture solution to biocement solution.

Centrifugation is also needed in the batch system. Usually, the 100 L culture solution is continuously centrifuged at 4 °C. The centrifugal concentration factors are usually 250 to 300. It took approximately 9 h to centrifuge the 100 L of the culture solution. A fifteen percent glycerin solution was added to the centrifuged microbes and mixed well. This was used to reduce damage to the microbes by freezing [29]. After mixing, the mixtures were packed in plastic bags or plastic tubes, and frozen in cooled ethanol containing the pieces of dry ice at below −40 °C for more than 20 min. The frozen mixtures were stored in a deep freezer at −80 °C until utilized, as illustrated in Figure 2.

Under this situation, the quality of stored microbes is usually unknown because of many governing factors, such as the varied nature in the original culture solution, i.e., inherent properties, centrifugal effects, amounts of microbes in the supernatant during centrifuging, effects of added glycerin, aging, thawing effects, etc. Most of these factors reduce the number of viable cells. Accordingly, the urease activity in the BC solution (BCS) will decrease. To characterize the properties of BCS for engineering purposes, it is required to establish a technique to determine the quality of microbes, including the carbonate precipitation rate (CPR). Herein, CPR is defined as the mass of precipitated carbonates, or the mass of precipitated carbonates for a given time. 

In this study, the standard CPR was obtained using OD related to cell viability. Next, the calibration curves were introduced from the standard CPR curve, assuming that OD = Rcv OD*. Herein, OD* was tentatively used as the calculated OD value for the BCS, calculated by the dilution factor from the concentrated volume of microbes. Ultimately, the CPR curves with OD* were checked against the calibration curves, and the Rcv value was obtained. These are discussed later.

When it is expressed as the precipitation rate of CaCO_3_, it is roughly divided into two meanings. One is to express the amount of precipitation by the quantity or percentage, and the other is to represent the precipitation rate. In this study, moles (M) were used to represent them by quantity, and percentages are used to represent them as a percentage. On the other hand, if it is expressed in the amount precipitated for a certain time, the precipitation speed was used.

**Figure 3:**
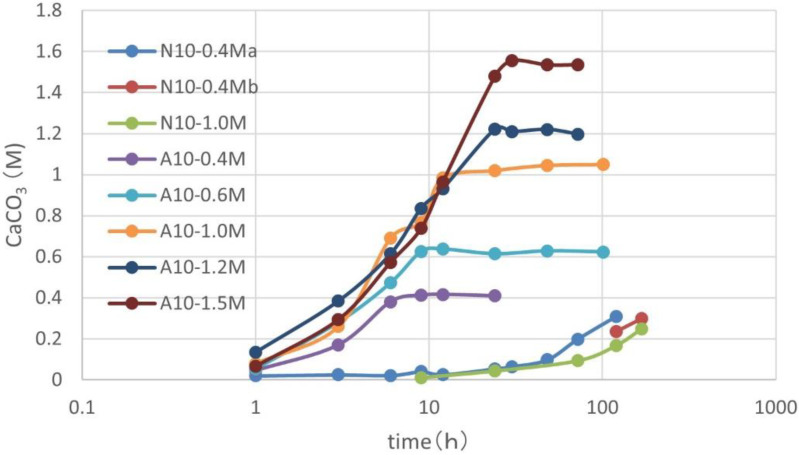
CPRs as a function of time for A10 and N10, under 1.0 OD and various Ca^2+^.

The fundamentals of CPR were studied using the fresh culture in the laboratory. CPRs induced by two ureolytic microbes using 1.0 OD and various Ca^2+^ concentrations are shown in Figure 3. The two types of microbes were named NO-N10 and NO-A10. These are expressed as N10 and A10, respectively. In Figure 3, A10 shows the rapid increases in CPR. It is obvious that the CPRs are controlled by Ca^2+^. Thus, the limitation of Ca^2+^ clearly indicates that the chemical reactions occurred according to Reactions (1), (2), and (3), with partial constraints. It is noted that no precipitation occurred if the concentration of Ca^2+^ exceeded 1.5 M [28].

On the other hand, for the case of N10, the reactions were not completed. This is because of low urease activity. The two CPRs curves for N10 are below the Ca^2+^ levels. Concerning the reaction rate, it is common for the lower Ca^2+^ levels to cause the higher precipitation rate. This is discussed later.

**Figure 4:**
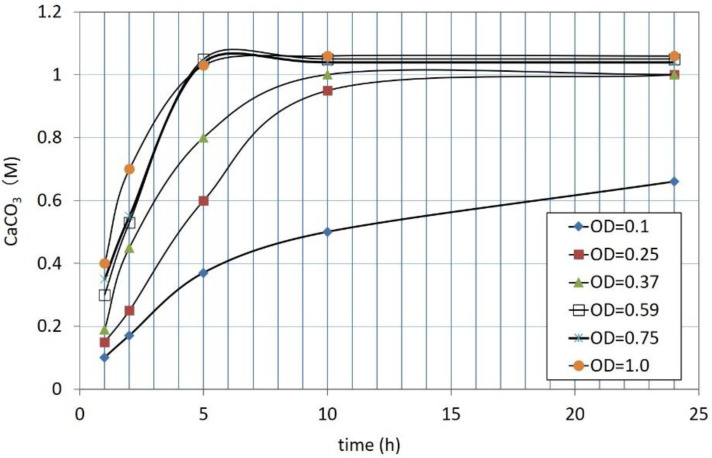
CPRs according to elapsed time for A10 strains using 1 M Ca^2+^ and various OD values.

The effects of OD using 1M Ca^2+^ are shown in Figure 4. The various OD values were prepared by dilution of the 1.0 OD solution. The initial pH value used was approximately 9.5 due to the 20 mM ammonium buffer. The ambient temperature was 25 °C. The result showed that the CPRs were rapid at the greater OD. If 24 h is selected as the measured time, all of the curves except for 1.0 OD show that the CPRs at 24 h are nearly 1.0 M or greater than that. It is noted that the CPRs greater than 1.0 M can be obtained with greater OD values. This is because hydrated carbonates tend to be produced when the reaction rate is high [30]. For the CPR curve for OD = 0.1, the CPR is 0.65 M.

### 2.2. Limitation of Urease Activity for Carbonate Precipitation

Considering that the hydrolysis of urea occurs inside of the cell, and that the ammonia and carbon dioxide react with water on the surface of the outside of the cell, molecular-sized carbonates will begin to cover the cell from the initial stage. Accordingly, the cell becomes a nucleus of the induced carbonate particles [31]. Okwadha and Li [32] concluded that the bacterial cell concentration, initial urea concentration, and Ca^2+^ concentration all influenced the amount of CaCO_3_ precipitated and CO_2_ sequestrated. Their results also indicated that a greater amount of CaCO_3_ would be precipitated with a greater concentration of urea, Ca^2+^, and bacterial cells, so long as these quantities are economically feasible.

In general, the enzyme is not a reactive material. Therefore, it is not consumed in the hydrolysis of urea. In this sense, the hydrolysis continues if urea is supplied from the outside of cell. On the other hand, CaCO_3_ precipitation continues around the surface of cell until Ca^2+^ is consumed or the supply of CO_2_ or CO_3_^2−^ or H_2_O stops in Reactions (1) to (3). This condition may be due to the CaCO_3_ amount per cell becoming too high and the precipitation of CaCO_3_ being inhibited by the excess CaCO_3_. Therefore, the hypothetical limitation of CaCO_3_ precipitation per cell must be considered.

Counting the number of cells is a difficult task; simple methods include the optical density (OD) measurement of the cell suspension [33,34]. OD is a fast, cheap, and high-throughput measurement method widely used to estimate the density of cells in liquid culture [34].

Assuming that all of the cells are viable and under constant Ca^2+^, the microbial CaCO_3_ precipitation can be written as a function of OD and time. If the precipitation rate is defined at a given constant time, the precipitation rate at a given time is expressed as a function of OD only as follows:

CaCO_3_ = f(OD) (4)

It is assumed that Equation (4) has a limitation, such as the upper limit of CaCO_3_ produced by unit cell. In this study, the limitations and interferences in Equation (4) are investigated experimentally. However, there is another problem: the cell viability reduces with age, temperature change (cold and heat shocks), freezing, and freezing–thawing cycles [29,35]. The drainage of the supernatant containing bacteria cells in centrifuging process causes a reduction in viable cells. These facts require a simple test method for obtaining OD values that can be a representative of the number of viable cells. Note that the conventional OD_600_ cannot reflect the viable quantity of cells of BCS to be used.

### 2.3. Preparation of Sample for Obtaining Standard Precipitation Rate

The relationship between the OD value and precipitation rate for CaCO_3_ was investigated based on the hypothesis described earlier. First, the OD of the fresh culture solution of A10 was adjusted to 1.0. The number of cells, i.e., colony forming unit (CFU), was measured using the agar culture technique. The cell viability corresponding to this CFU was assumed to be 100%. The solution was divided into three 100 mL flasks. The solution in the first flask was immediately used as fresh samples with 100% viability. Second flask was stored in the refrigerator at 4 °C for one month, and the CFU was measured. Comparing the CFU value with that of 100% viability, the viability was estimated as 45%. Assuming the linear relationship between cell viability and OD value, the OD of the second solution was determined as 0.45. The third flask was kept at room temperature, 25 °C, for one month. Like the second solution, CFU, viability, and OD were determined in order. As a result, the viability and OD for the third solution were 9% and 0.09, respectively. These three types of solutions were used for the following precipitation tests as the undiluted BCSs, respectively.

The samples for precipitation tests were prepared as follows.

For preparation of the OD value, the prescribed OD values were arranged in 10 mL BCS in test tubes, as indicated in Table 1. To carry this out, each 10 mL bio-solution was centrifuged using a centrifuge tube, and the supernatant was removed.

For the reactive solution,

(1) Prescribed quantities of reagents, i.e., urea, calcium chloride, etc., were prepared;
(2) Ten milliliter reactive solutions were added to each test tube containing the centrifuged microbes. Note that the volume of microbes is negligible, as the centrifugal concentration factor by volume is approximately 250–300;
(3) The test tubes were left for 24 h, and the precipitated CaCO_3_ was measured by dividing into suspended particles and adsorbed carbonates.

**Figure 5:**
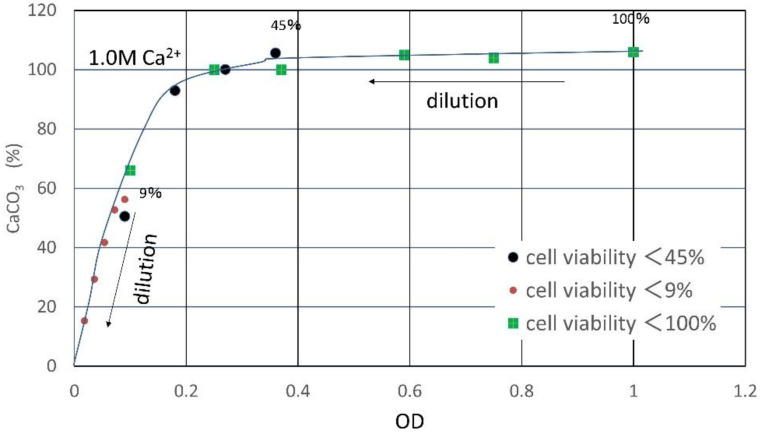
Relationship between precipitation rates and OD related to cell viability, showing a standard precipitation curve.

The results are indicated in terms of the carbonate precipitation rate (CPR) in Table 1 and Figure 5. It is seen that the quality of microbe changes with temperature, age, or dilution. In Table 1, it is seen that the precipitates for some samples exceed 1.0 M. This is possibly due to the precipitation of hydrated amorphous calcium carbonates (ACC) [30]. This unique relationship was obtained for the OD values under the controlled cell viability as the best performance. If the quality of microbes vary, the plots might deviate widely. 

In this study, the CPR of the unspecified BCS was examined in terms of the estimated OD, which was calculated using only dilution effect. The line shown is defined as the standard precipitation curve, which is given as follows:

CaCO_3_ = CPR = 8.460 OD − 17.633 (OD)^2^   (M) (5)

**Figure 6:**
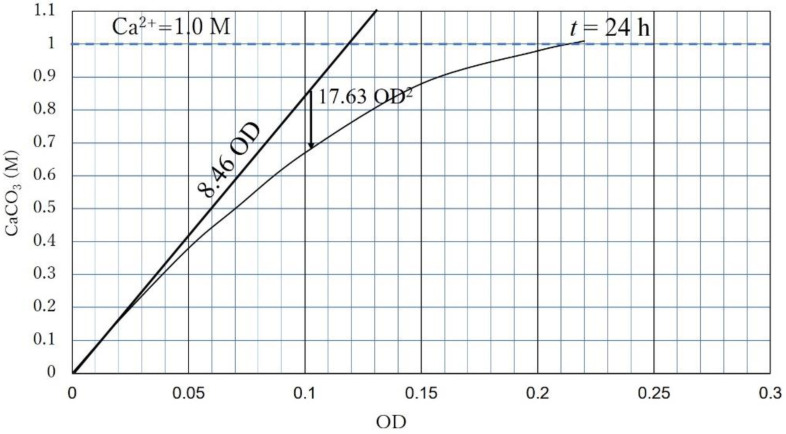
Illustration of Equation (5).

Equation (5) is a quadratic function of OD showing a convex at the top, and is illustrated in Figure 6. It seems that the first order term on the right side of Equation (5) shows the CaCO_3_ precipitation rate with somewhat inherent properties of microbes, such as the activity of the unit cell. On the other hand, the second order term may be related to the interaction between microbes and Ca^2+^, and the competition of microbes. It may cause the delayed reaction or constrained reaction.

### 2.4. Calibration Curves

Considering the reduction in cell viability, the OD value is assumed as follows:

OD = Rcv OD*(6)

where Rcv is the conversion factor and OD* is the tentative optical density associated with the dilution factor used for sample preparation. The OD values of the test samples can be varied by diluting an undiluted BCS, which concerns the number of viable cells. Using the dilution technique, the CPR is measured in terms of various OD* values. Introducing Equation (6) into Equation (5), the CPR as a function of OD* and Rcv is as follows:

CaCO_3_ = 8.460 Rcv OD* − 17.633 (Rcv OD*)^2^   (M) (7)

**Figure 7:**
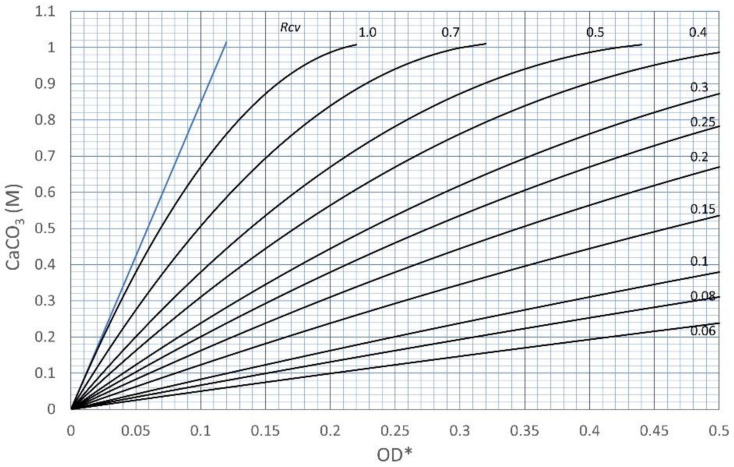
Calibration curves in terms of OD* and CPR with various Rcv values.

In Equation (7), if the induced CaCO_3_ is given, the value of Rcv OD* can be determined using the quadric formula. Therefore, if the OD* is given, the corresponding Rcv is also determined. Experimentally, if the relationship between CPR and OD* is obtained, the corresponding Rcv is determined by fitting the experimental data with calibration curves. The calibration curves consist of CPR-OD* relationships with a constant Rcv value, as shown in Figure 7. In general, the OD* value is given as an arbitrary experimental condition, provided that, in this study, it is defined according to a certain rule. The rule is needed to make Rcv meaningful, and is defined later.

Since the various curves with different Rcv values are derived from the origin of OD* and CaCO_3_ axes, the curves do not cross. Therefore, any point in the curve’s area on the coordinate has its own Rcv value.

### 2.5. Materials

Materials used were microbes A10 strains, urea, calcium chloride, ammonia buffer solution, and water as solvent. The optimum blending of these materials is under consideration. Various ages of microbes were used to examine the ageing effects of microbes. The longest frozen time used was 1 year at −80 °C. The aspects of the samples were assumed as unknown, and the parameters used were the initial optical density of the culture solution of microbes, ODi, and concentration factor, Cf.

### 2.6. Methods

The volume of microbes, Vmc, to be used was calculated as follows:

Vmc = (Vbc OD*)/(ODi Df)(8)

where the initial optical density ODi and dilution factor Df (=concentration factor Cf) are known, and the volume of BCS Vbc and desired tentative OD* are given. For example, when 100 mL BCS with 1.28 OD* is required, the volume of microbes Vmc is needed as

Vmc = (100) (1.28)/{(4.0) (250)} = 0.128   (mL)(9)

where ODi is usually 4.0, and Cf and Df are usually around 250, as shown in Equation (9). If Vmc was a very small amount, it was converted to the mass using a density of the concentrated microbes, i.e., 1.07 g/cm^3^. Then, the microbes were weighed.

## 3. Results and Discussion

### 3.1. CPR Controlled by Ca^2+^

If the BC solution was prepared with a relatively large density of viable cells, the CPR can be controlled by the Ca^2+^ used. The hydrolysis of urea is completed by the microbe’s enzymes, and the carbonate ions required will be produced. Accordingly, Reactions (1) and (2) occur, provided that the Ca^2+^ is arbitrary. This reaction process is easily examined using BCS. This is confirmed by the controlled precipitation by Ca^2+^ as follows.

A hundred milliliters of BCS prepared by Equation (8) can be used for more than ten specimens with 10 mL. For the precipitation tests, the combination of Ca^2+^ and OD* is selective. For a typical example, the BC solution with 1.0 M Ca^2+^ and 1.0 OD can be diluted with dilution factors of 0.7, 0.5, 0.3, and 0.1, respectively. Distilled water or a saline solution was used for dilution. In the case of this dilution, the prepared BC solutions have the same Ca^2+^/OD* ratio, i.e., 0.78 M, and both Ca^2+^ and OD* decrease with the similar dilution factors. 

**Figure 8:**
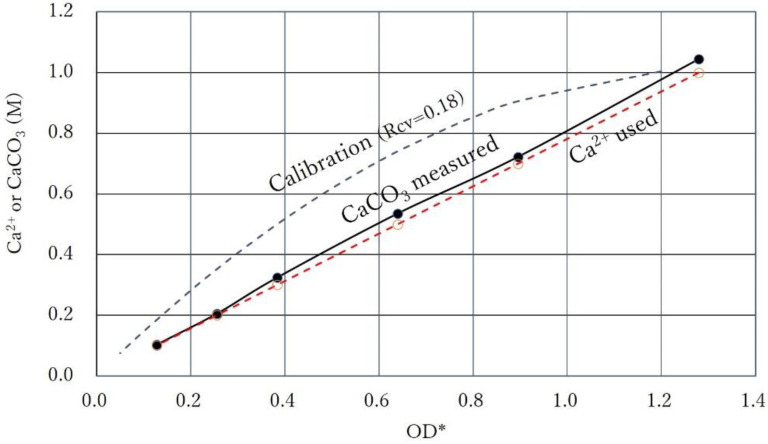
CPR controlled by Ca^2+^ levels, which shows the fact that the Ca^2+^ used was all consumed, and the calibration curve with minimum Rcv required for obtaining the results of precipitation tests (at 24 h).

The used Ca^2+^-OD* and the induced CaCO_3_-OD* relationships are shown in Figure 8, which clearly indicates that the two relationships are parallel straight lines. This indicates that the CPRs were controlled by Ca^2+^. In Figure 8, the molar concentrations of the induced CaCO_3_ are slightly higher than that of Ca^2+^. This is due to the hydration of carbonates, as described earlier. If the carbonate precipitations for all samples occurred under control of Ca^2+^, it means that the number of cells was sufficient to complete the reactions. Accordingly, the Rcv values for the BCSs cannot be specified, but the minimum Rcv is evaluated, as shown in the Figure. It indicates that, if BCS has a Rcv value greater than 0.18, the precipitation result is like Figure 8. Thus, in those cases, a proper Rcv cannot be evaluated. However, in the design of the blending of BCS, the minimum value of Rcv provides significant information.

**Figure 9:**
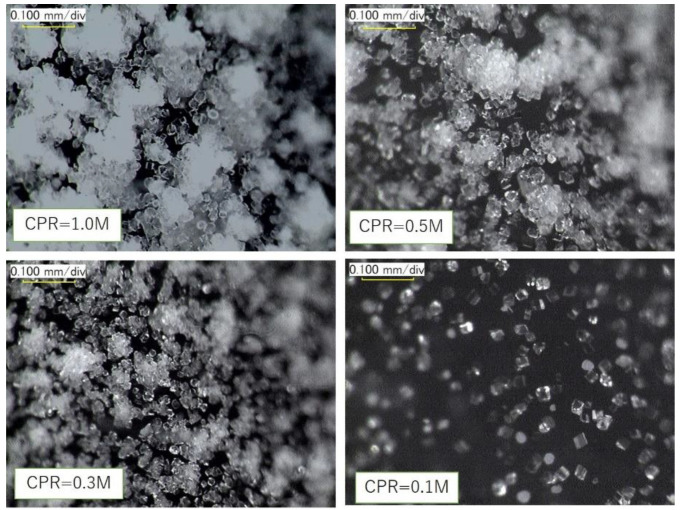
Digital photomicrographs of precipitated CaCO_3_ (at 24 h) on the walls of test tubes, using various levels of Ca^2+^.

In Figure 9, microscopic photos for 1.0, 0.5, 0.3, and 0.1 M CaCO_3_ corresponding to Figure 8 are demonstrated. The calcium carbonates fully precipitated on the inner walls of the test tubes were dependent on the used levels of Ca^2+^. The precipitation time was approximately 24 h. It is noted that, in general, the minerals precipitated are not always the same, but vary with temperature, pH, reaction rates, etc. In general, minerals induced by microbial precipitation are hydrated amorphous calcite (ACC), calcite, aragonite, vaterite, etc. [36,37], whose size and shape (crystals) often vary with time. However, it was reported that microbes and urease do not affect the morphology of carbonates to be induced [38]. This may be true because microbes become nuclei for carbonate formation and urease only has a role as an enzyme for catalysis in the hydrolysis of urea. There is no direct evidence that microbes and urease affect the morphology of carbonates.

It was confirmed that these particles are dissolved by 3N HCl. The carbonate content was measured by mass analysis using HCl. The particles were also observed by a microscope as amorphous or crystalline calcites, and vaterite. Calcite is known to exhibit many morphological aspects, i.e., many crystalline shapes.

### 3.2. Effects of Increasing Ca^2+^/OD*

**Figure 10:**
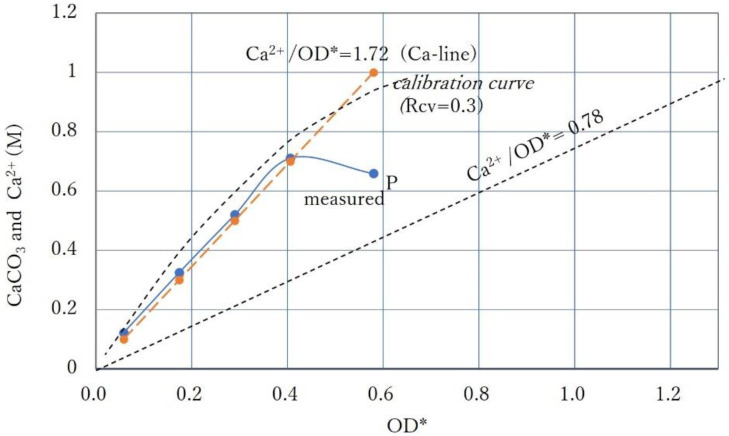
CPR curve for a relatively high Ca^2+^/OD* level and calibration curve evaluated with Rcv = 0.3.

For the next tests, the Ca^2+^/OD* ratio was increased to 1.72. The BCSs used were diluted with factors of 0.7, 0.5, 0.3, and 0.1. The results of the precipitation tests are shown in Figure 10. It shows that, in comparison to Figure 8, the higher precipitation occurred for the higher Ca^2+^/OD* ratio. However, the CPR at the point P for OD* = 0.58 was lower than expected. It is considered that the precipitation indicated by point P at OD* = 0.58 was delayed because the number of cells was not enough under 1.0 M Ca^2+^. This is examined later.

The CPRs for the OD* lower than 0.4 are expressed by a straight line, so the precipitation was probably controlled by the Ca^2+^ used. Therefore, the calibration curve for this case can be fitted by Rcv = 0.3, as shown in Figure 10. It is a key point in this case that the calibration curve passes between the Ca^2+^ used and the lower measured CPR at a given OD* value. Note that the OD is evaluated by 0.3 OD* using Rcv = 0.3 because of the definition in Equation (6). Therefore, if 0.4 OD*, i.e., 0.12 OD is used, it is found that approximately 0.7 M CaCO_3_ is obtained.

**Figure 11:**
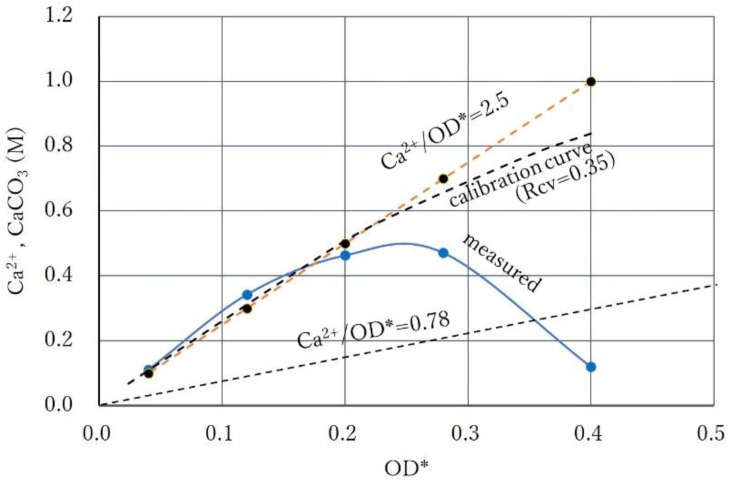
Relatively high CPRs at lower OD * and over-loading effects at higher OD * under the condition of relatively high Ca^2+^/OD *.

To see this phenomenon in detail, the Ca^2+^/OD* ratio was increased again. The results of the precipitation tests at a Ca^2+^/OD* ratio of 2.5 is indicated in Figure 11. It shows the relatively high CPR below OD* = 0.2 and the inhibitory effect at a higher OD*. The CPR at OD* = 0.4 was only 12% of the applied Ca^2+^(=1.0 M). This decreasing effect is possibly due to the shape of the calibration curve depending on the second order term of OD in Equation (5) or (7). In fact, this second order term can be understood as the delayed reaction from 24 h for the growth of mineral grains.

The effect of the delayed reaction on the engineering properties of biocement has not been well studied. It may give good results in terms of cementation effects. However, considering the various cases in the lab and field conditions, the inhibitory phenomena of CPR with Ca^2+^ is defined as the over-loading effect and is distinguished from the standard precipitation rate in this study.

In Figure 11, the over-loading effect also seems to occur at OD* = 0.28. Thus, it is found that, with increasing Ca^2+^/OD*, the CPR increases at a lower OD*, whereas the over-loading effect occurs at a higher OD*.

### 3.3. Over-Loading Effect

**Figure 12:**
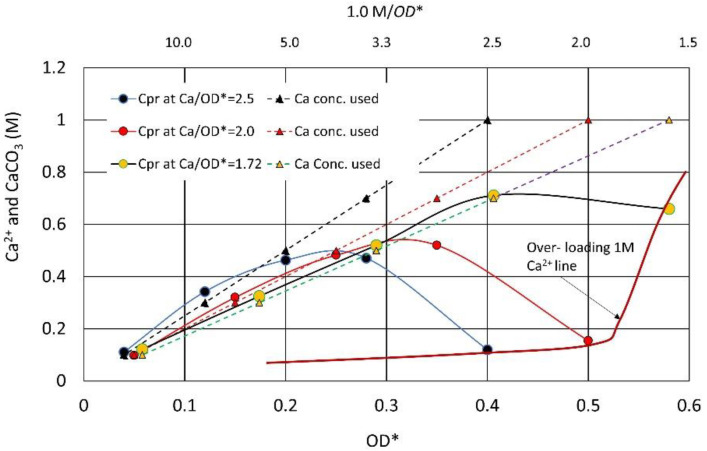
Over-loading effects due to relatively high Ca^2+^.

In Figure 12, the CPRs for the highest OD* values at various Ca^2+^/OD* ratios are indicated as the over-loading 1M Ca^2+^ line. It indicates that higher Ca^2+^ causes the over-loading effect. On the other hand, for the lower OD*, a relatively high CPR corresponding to the Ca^2+^ used is obtained.

### 3.4. Over-loading Effects for Low OD Values 

**Figure 13:**
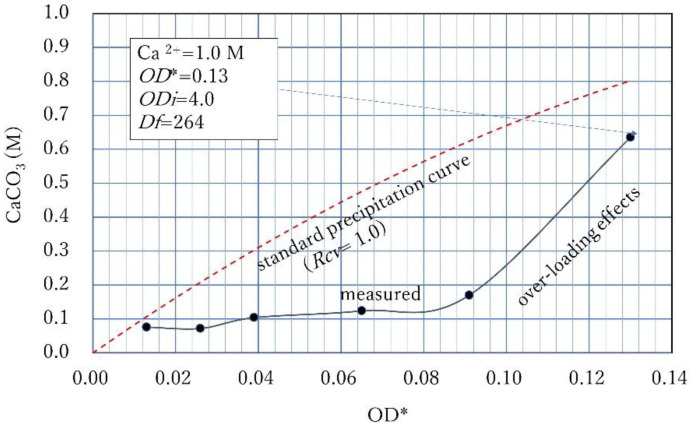
Over-loading effect under high Ca^2+^/OD* ratios, where OD*≒OD.

If the OD value is unknown, it is not known if the over-loading effect occurs. Another example of the over-loading effect is obtained using different samples, as shown in Figure 13. The test conditions are presented in terms of Ca^2+^, OD*, ODi, and Df for the undiluted BCS. The undiluted OD* value used is already adjusted to the OD value. Therefore, the diluted OD* values are also equivalent to the OD value. The calculated Ca^2+^/OD ratios for the specimens ranged from 7.7 M to 83 M. The result is very similar to that shown in Figure 12. Thus, the results assured the repeatability of the over-loading effect. If the over-loading effect is known, lower Ca^2+^ levels are selected, i.e., the CPR of the standard precipitation curve can be obtained in 24 h.

### 3.5. Intersection of Constant Ca^2+^ Line and Calibration Curve 

**Figure 14:**
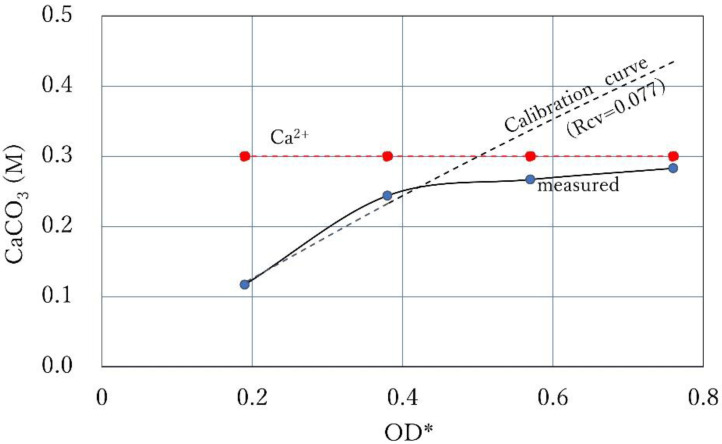
CPR controlled by 0.3 M Ca^2+^ and the calibration curve with a Rcv of 0.077.

It was described earlier that the CPR cannot exceed the Ca^2+^ line, except for the occurrence of hydrated amorphous carbonate (ACC). Figure 14 shows the case where the constant Ca^2+^ line intersects with the calibration curve. The CPR line at 24 h increases along the calibration curve (Rcv of 0.077) from the lower OD* value until it is controlled by Ca^2+^, as shown in Figure 14. Near the Ca^2+^ line, the CPR is controlled by the amount of Ca^2+^. If ACC forms, the CPR may exceed the Ca^2+^ line because of the effect of hydration, as was described. It is noted that the OD values are estimated by Equation (6), i.e., Rcv OD *.

### 3.6. Example of Translation from CPR-OD* to CPR-OD Relations

**Figure 15:**
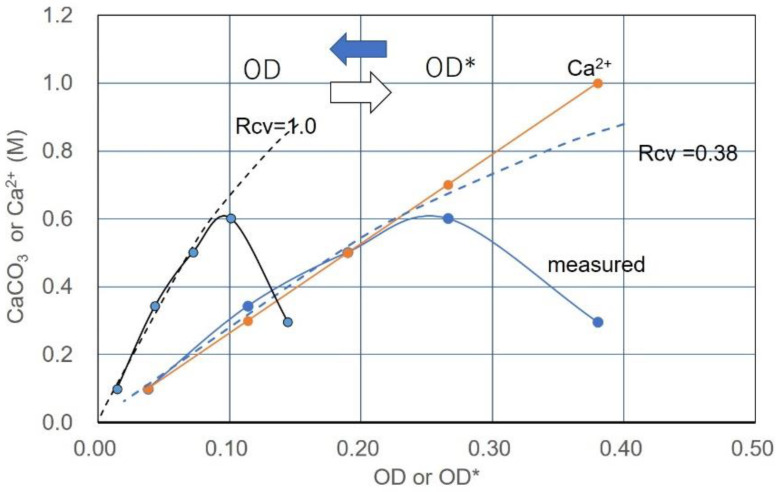
Conversion from OD* to OD or OD to OD* using Rcv value estimated.

If the Rcv value is obtained, the corresponding OD value is determined from Equation (6). The conversion from OD* to OD or OD to OD* is shown in Figure 15. The horizontal axis is used for both the OD and OD* values. As can be seen, the precipitation rates were converted by the coordinate conversion from OD* to OD. The agreement between the standard curve and the converted points of the precipitation rates indicates that the value of the selected Rcv was appropriate. It shows that, if the molar concentration of used Ca^2+^ is greater than that of CaCO_3_ given by the standard curve, the actual precipitation of CaCO_3_ will decrease from the value predicted by the calibration, as shown in the figure. This is discussed later.

### 3.7. CPR after Over-Loading Effects

To investigate time dependence on the over-loading effect, the precipitation rates were measured at a longer elapsed time, under the condition of 1.0 M Ca^2+^. The test conditions used are indicated in Table 2. Note that the No. 1 specimen consisted of five aliquots, whereas four aliquots were used for No. 2. The CaCO_3_ measurements of each sample were made at a different date. Herein, CPR is defined for CaCO_3_ precipitation at any elapsed time. However, it does not mean the precipitation rate, but the amount.

**Figure 16:**
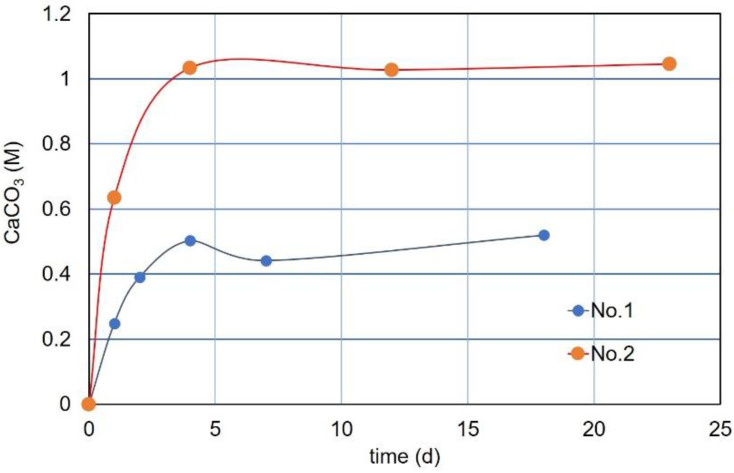
Long time precipitation patterns of CaCO_3_ obtained by aliquot samples.

The precipitated amounts measured are shown in Figure 16. The precipitation seems to end at certain Ca^2+^ values. For the No. 1 specimen, the precipitation rate at 24 h was approximately 0.25 M, which was approximately a half of the standard value. The CPR increased by approximately 0.5 M at 4 days. After 18 days, the precipitation decreased to approximately 0.45 M but slightly increased again, as shown in Figure 16.

For the No. 2 specimen (four aliquots), the precipitation rate was approximately 0.63 M at 24 h. The value is smaller than the standard at 24 h. The precipitation exceeded 1.0 M 4 days after and then decreased 12 days after. However, it seems to be slightly increased again, as shown in Figure 16. The decrease in volume of CaCO_3_ after a few days is common. It may be due to the metamorphism of carbonate crystals, which may be due to the dehydration of hydrated carbonates. It is considered that such a slight change in CaCO_3_ precipitation may be due to the combined effects of hydration, dehydration, metamorphism, delayed formation of carbonates, etc.

**Figure 17:**
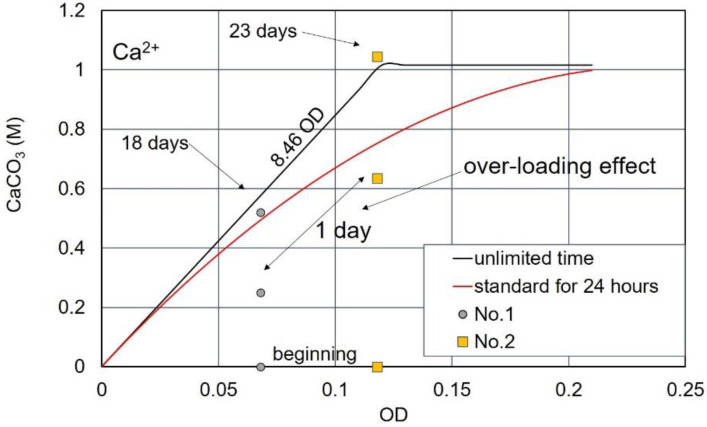
Precipitation rates after the over-loading effect of Ca^2+^, showing that ultimate precipitation of carbonates can be presented as a linear function of OD value.

For specimens No.1 with an OD value of 0.068, the 24 h precipitation was approximately half that of the standard curve at 24 h, but exceeded the standard curve after 4 days. The measured CaCO_3_ after 18 days is plotted as shown in Figure 17. For the No. 2 specimen, the precipitation at 0.12 OD exceeded the standard curve (24 h) on the second day. After 3 days, the CaCO_3_ production reached almost 1.0 M. The measured value of 1.03 M after 23 days is plotted, as shown in Figure 17.

From the observation of a long time CaCO_3_ precipitation, it was found that the amount of CaCO_3_ exceeded the standard value. Therefore, the second term of the right side in Equation (5) is considered to be a delayed effect due to the over-loading effect of Ca^2+^. It means that, given the unlimited time, the second term limited by 24 h can be ignored. As a result, the standard curve without time limitation is represented by a linear relationship of the following equation, leaving only the first term.

CaCO_3_ = 8.46 OD   (M) (10)

Equation (10) indicates that the maximum amount of precipitation is proportional only to the OD value (representative of the viable cell number). It was a hypothetical starting point in this study on the blending design of bio-cement.

## 4. Conclusions

The carbonate precipitation induced by ureolytic microbes was investigated to develop the optimum engineered blending method of BCS. Based on the hypothesis that CPR is governed by OD with viable cells, the unique standard precipitation curve for A10 strains, a basic calibration curve was determined. To apply the unique standard precipitation curve, the tentative OD* value was defined. The OD* is associated with OD = Rcv OD*, where Rcv is defined as the conversion factor concerned with cell viability. Herein, the CPR- OD* relationship can be converted to the CPR-OD relationship in terms of Rcv. This concept was experimentally examined. As a result, it was found that Rcv is predictable compared to the unique standard CPR-OD and the experimental CPR-OD* relationship. It was also found that the OD value was predictable using Rcv and OD*. With this, it became possible to evaluate the quality of microbes uniformly, without measuring OD_600_. Furthermore, multiple achievements were obtained through this study. The main achievements are summarized as follows.

(1) The microbial strain (NO-A10) is mainly examined in this study. Note that the quantitative results presented are dependent on microbes and strains;
(2) The CPR desired can be blended in terms of OD and Ca^2+^;
(3) CPR is governed by OD or Ca^2+^. If the OD is dominant, the CPR (24 h) is expressed as CPR = 8460 OD − 17.633 OD^2^;
(4) If Ca^2+^ is dominant, CPR= *a* CaCO_3_ (M/24h) for the case of NO-A10 strains and *a* M Ca^2+^;
(5) If Ca^2+^ is high and OD is relatively low, the over-loading effect, which causes exceptionally low CPR, occurs. However, the carbonate precipitation increases according to the following extension of the concept with time;
(6) Extending the concept of the unique standard precipitation rate regarding time, the CPR-OD relationship was simply expressed by CPR = l OD, where l was 8.46 M for NO-A10 strains. Note that the CPR does not necessarily mean CaCO_3_ precipitated for 24 h.

The study is based on the BCS, but not on the soil-BCS system. However, the microscopic photos presented in this paper indicate the carbonate precipitated on the surfaces of glass like minerals. The effect of the adsorption between microbes and soil particles may influence the Ca^2+^ and OD * values. It may depend on the soil depth or flow distance associated with the injection of the solution. Accordingly, the adsorption properties and behavior of Ca^2+^ and microbes on soil particles can be important for future study. The most important contribution in this research is the determination that CPR is controlled by the terms of Rcv, OD *, and Ca^2+^.

## Figures and Tables

**Table 1:** Experimental data for determination of a standard precipitation curve.

Flask No.	Temp. (°C)	Age	Spec. No.	Cell Viability (%)	OD	CPR (M)
1	25	1 day	1	**100**	**1.0**	1.06
			2		0.75	1.04
			3		0.59	1.05
			4		0.37	1.00
			5		0.25	1.00
			6		0.10	0.66
2	4	1 month	7	**45**	**0.45**	—
			8		0.36	1.06
			9		0.27	1.00
			10		0.18	0.93
			11		0.09	0.51
3	25	1 month	12	**9**	**0.09**	0.56
			13		0.072	0.53
			14		0.054	0.42
			15		0.036	0.29
			16		0.018	0.15

**Table 2:** Precipitation tests after over-loading effects.

No.	OD*	Rcv	OD	Ca^2+^	Ca^2+^/OD*	Ca^2+^/OD
1	0.180	0.38	0.068	1.0	5.56	14.62
2	0.310	0.38	0.118	1.0	3.23	8.47

## Data Availability

Data available on request.

## References

[1] Friedman G.M. (1998). Rapidity of marine carbonate cementation-implications for carbonate diagenesis and sequence stratigraphy: Perspective. Sed. Geol..

[2] Fukue M., Nakamura T., Kato Y., Naoe K. (1996). Correlation among carbonate content, accumulation rate and topography of seabed. Soils Found..

[3] Fukue M., Nakamura T., Kato Y. (1999). Cementation of soils due to calcium carbonate. Soils Found..

[4] Moore C.H. (2001). Developments in Sedimentology. Carbonate Reservoirs, Porosity Evolution and Diagenesis in a Sequence Stratigraphic Framework.

[5] Castanier S., Métayer-Levrel G.L., Perthuisot J.-P. (1999). Ca-carbonates precipitation and limestone genesis-the microbiogeologist point of view. Sed. Geol..

[6] Neumeier U. (1999). Experimental modelling of beachrock cementation under microbial influence. Sed. Geol..

[7] Mobley H.L.T., Hausinger R.P. (1989). Microbial urease: Significance, regulation, and molecular characterization. Microbiol. Rev..

[8] Fenn L.B., Miyamoto S. (1981). Ammonia loss and associated reactions of urea in calcareous soils. Soil. Sci. Soc. Am. J..

[9] Akiyama M., Kawasaki S. (2019). Biogeochemical simulation of microbially induced calcite precipitation with *Pararhodobacter* sp. strain SO1. Acta Geotechnol..

[10] Azadi M., Ghayoomi M., Sgamskia N., Kalantari H. (2017). Physical and mechanical properties of reconstructed bio-cemented sand. Soils Found..

[11] Cheng L., Shahin M., Chu J. (2019). Soil bio-cementation using a new one-phase low-pH injection method. Acta Geotechnol..

[12] Jiang N.-J., Soga K. (2019). Erosional behavior of gravel-sand mixtures stabilized by microbially induced calcite precipitation (MICP). Soils Found..

[13] Kahani M., Kalantary F., Soudi M.R., Pakdel L., Aghaalizadeh S. (2020). Optimization of cost-effective culture medium for *Sporosarcina pasteurii* as biocementing agent using response surface methodology, Up cycling dairy waste and seawater. J. Clean. Prod..

[14] Lin H., Suleiman M., Jabbour H.M., Brown D. (2018). Bio-grouting to enhance axial pull-out response of pervious concrete ground improvement piles. Can. Geotechnol. J..

[15] Sun X., Miao L., Tong T., Wong C. (2019). Study of the effect of temperature on microbially induced carbonate precipitation. Acta Geotechnol..

[16] Kim D., Park K., Kim D. (2014). Effects of Ground Conditions on Microbial Cementation in Soils. Materials.

[17] Chen M., Gowthaman S., Nakashima K., Komatsu S., Kawasaki S. (2021). Experimental Study on Sand Stabilization Using Bio-Cementation with Wastepaper Fiber Integration. Materials.

[18] Konstantinou C., Biscontin G., Logothetis F. (2021). Tensile Strength of Artificially Cemented Sandstone Generated via Microbially Induced Carbonate Precipitation. Materials.

[19] Cheng L., Shahin M.A., Mujah D. (2016). Influence of key environmental conditions on microbially induced cementation for soil stabilization. J. Geotechnol. Geoenviron. Eng..

[20] Van Paassen L.A. (2009). Biogrout, Ground Improvement by Microbial Induced Carbonate Precipitation. Ph.D. Thesis.

[21] DeJong J.T., Fritzges M.B., Nüsslein K. (2006). Microbially induced cementation to control sand response to undrained shear. J. Geotechnol. Geoenviron. Eng..

[22] DeJong J.T., Mortensen B.M., Martinez B.C., Nelson D.C. (2010). Bio-mediated soil improvement. Ecol. Eng..

[23] Fukue M., Lechowicz Z. Strength of biocemented sandy soils using simple devices. Proceedings of the International Congress Natural Sciences and Engineering.

[24] Choi S.-G., Chang I., Lee M., Lee J.-H., Han J.-T., Kwon T.-H. (2020). Review on geotechnical engineering properties of sands treated by microbially induced calcium carbonate precipitation (MICP) and biopolymers. Constr. Build. Mater..

[25] Benini S., Gessa C., Ciurli S. (1996). *Bacillus Pasteurii* urease: A Heteropolymeric enzyme with a binuclear nickel active site. Soil Biol. Biochem..

[26] Omoregie A.I., Khoshdelnezamiha G., Senian N., Ong D.E.L., Nissom P.M. (2017). Experimental optimization of various cultural conditions on urease activity for isolated *Sporosarcina pasteurii* strains and evaluation of their biocement potentials. Ecol. Eng..

[27] Omoregie A.I., Ginjom R.H., Nissom P.M. (2018). Microbially induced carbonate precipitation via ureolysis process: A Mini- Review. Trans. Sci. Technol..

[28] Fukue M., Ono S., Sato Y. (2011). Cementation of sands due to Microbiologically induced Carbonate. Soils Found..

[29] Nakamura M., Farnum J.L., Oke M.A. (1962). Protective action of Glycerol in the freezing of *Shigella sonnel*. Nature.

[30] Rodriguez-Blanco J.D., Sand K.K., Benning L.G., Van Driessche A., Kellermeier M., Benning L., Gebauer D. (2017). ACC and vaterite as intermediates in the solution-based crystallization of CaCO_3_. New Perspectives on Mineral Nucleation and Growth: Chapter 5.

[31] Stocks-Fischer S., Galinat J.K., Bang S.S. (1999). Microbiological precipitation of CaCO_3_. Soil Biol. Biochem..

[32] Okwadha G.D.O., Li J. (2010). Technical Note: Optimum conditions for microbial carbonate precipitation. Chemosphere.

[33] Francois K., Devlieghere F., Standaert A.R., Geeraerd A.H., Cools I., Van Impe J.F., Debevere J. (2005). Environmental factors influencing the relationship between optical density and cell count for *Listeria monocytogenes*. J. Appl. Microbiol..

[34] Beal J., Farny N.G., Haddock-Angelli T., Selvarajah V., Baldwin G.S., Buckley-Taylor R., Gershater M., Kiga D., Marken J., Sanchania V. (2019). the iGEM Interlab Study Contributors, Robust estimation of bacterial cell count from optical density. bioRxiv.

[35] Garcia S., Limón J.C., Heredia N.L. (2001). Cross protection by heat and cold shock to lethal temperatures in clostridium perfringens. Brazil. J. Microbiol..

[36] Rodriguez-Navarro C., Jimenez-Lopez C., Rodriguez-Navarro A., Gonzalez-Muñoz M.T., Rodriguez-Gallego M. (2007). Bacterially mediated mineralization of vaterite. Geochim. Cosmochim. Acta.

[37] Wang Y., Yao Q., Zhoui G., Fu S. (2015). Transformation of amorphous calcium carbonate into monohydrocalcite in aqueous solution: A biomimetic mineralization study. Eur. J. Mineral.

[38] Wen K., Yang L., Farshad A., Lin L. (2020). Impact of bacteria and urease concentration on precipitation kinetics and crystal morphology of calcium carbonate. Acta Geotechnol..

